# Clinical impact of suboptimal RAASi therapy following an episode of hyperkalemia

**DOI:** 10.1186/s12882-022-03054-5

**Published:** 2023-01-19

**Authors:** Eiichiro Kanda, Anjay Rastogi, Toyoaki Murohara, Eva Lesén, Abiy Agiro, Matthew Arnold, Gengshi Chen, Toshitaka Yajima, Krister Järbrink, Charles V. Pollack

**Affiliations:** 1 Department of Medical Science, Kawasaki Medical School, 577 Matsushima, Kurashiki, Okayama, 701-0192 Japan; 2 Department of Medicine, David Geffen School of Medicine at the University of California Los Angeles, Los Angeles, CA USA; 3 Department of Cardiology, Nagoya University Graduate School of Medicine, Nagoya, Japan; 4 Medical & Payer Evidence, Cardiovascular, Renal and Metabolism, BioPharmaceuticals R&D, AstraZeneca, Gothenburg, Sweden; 5 US Evidence, Medical Affairs, BioPharmaceuticals R&D, AstraZeneca, DE Wilmington, USA; 6 Real World Science and Digital, AstraZeneca, Cambridge, UK; 7 Health Economics & Payer Evidence, AstraZeneca, Cambridge, UK; 8 CVRM TA, Medical Affairs, AstraZeneca, Tokyo, Japan; 9 Department of Emergency Medicine, University of Mississippi School of Medicine, Jackson, MS USA

**Keywords:** Hyperkalemia, Heart failure, Chronic kidney disease, Guideline-directed medical therapy, Potassium binder

## Abstract

**Background:**

Hyperkalemia (HK) is a barrier to optimization of renin-angiotensin-aldosterone system inhibitor (RAASi) therapy in heart failure (HF) and chronic kidney disease (CKD). We investigated cardiorenal risk associated with changes in RAASi regimen after an episode of HK in patients with HF and/or CKD.

**Methods:**

This observational study utilized data from hospital records, claims, and health registers from the US (Optum’s de-identified Market Clarity Data) and Japan (Medical Data Vision). Included patients had an index episode of HK between July 2019 and September 2021 (US), or May 2020 and September 2021 (Japan), with prior diagnosis of HF or CKD (stage 3 or 4), and RAASi use. Risk of a cardiorenal composite outcome (HF emergency visit, HF hospitalization, or progression to end-stage kidney disease) was determined in patients who discontinued RAASi, down-titrated their dose by > 25%, or maintained or up-titrated their dose following the HK episode.

**Results:**

A total of 15,488 and 6020 patients were included from the US and Japan, respectively. Prior to the episode of HK, 59% (US) and 27% (Japan) of patients had achieved > 50% target RAASi dose. Following the episode of HK, 33% (US) and 32% (Japan) of patients did not fill a new RAASi prescription. Risk of the cardiorenal outcome at 6 months was higher in patients who discontinued or down-titrated versus maintained or up-titrated RAASi treatment both in the US (17.5, 18.3, and 10.6%; *p* <  0.001) and in Japan (19.7, 20.0, and 15.1%; *p* <  0.001).

**Conclusion:**

HK-related RAASi discontinuation or down-titration was associated with higher risk of cardiorenal events versus maintained or up-titrated RAASi.

**Supplementary Information:**

The online version contains supplementary material available at 10.1186/s12882-022-03054-5.

## Background

Renin-angiotensin-aldosterone system inhibitors (RAASis) have been shown to reduce blood pressure and proteinuria [1], delay estimated glomerular filtration rate (eGFR) decline [2], and lower the risk of kidney failure, cardiovascular morbidity, and all-cause mortality in patients with chronic kidney disease (CKD) [2]. They also decrease cardiovascular morbidity and mortality in patients with heart failure (HF) [3]. Evidence-based guidelines for medical therapy for CKD and HF support the use of RAASis at the maximum tolerated dose to achieve optimal treatment benefits [4–9]. However, RAASis also increase the risk of hyperkalemia (HK), mediated by blockade of the RAAS and reducing renal potassium excretion. HK is an electrolyte disorder characterized by elevated levels of serum potassium (sK^+^) [10]. If left untreated, HK can lead to fatal cardiac arrhythmias, cardiac arrest, and sudden death [11, 12]. In a population-based cohort study, 28% of patients with CKD and 39% of patients with HF had at least one episode of HK, which occurred at a median of 1.2 and 0.6 years from the first CKD or HF diagnosis, respectively [13]. In addition, 40% of CKD and 49% of HF patients had a recurrent episode of HK within 6 months. A meta-analysis found that RAASi treatment was associated with a 2-fold higher risk of HK compared with control groups not receiving RAASis [14].

Despite strong evidence for the cardiorenal protective effects of RAASis, findings from observational studies of routine clinical practice show that prescribing RAASi doses below the maximum target dose is common in patients with HF [15–18] and CKD [16, 18–20]. A large European study found that while 67–92% of hospitalized patients with HF were treated with the recommended RAASi agents, two-thirds had not achieved the recommended target dose of those agents [21]. Other studies have demonstrated that therapy with RAASis below the maximum dose increases the risk of major adverse cardiac events and mortality in both CKD and HF populations [22, 23].

Current guidelines posit that HK should not be a barrier to RAASi optimization, but studies show that once RAASi is discontinued or down-titrated following an episode of HK, it is often not restarted or up-titrated [22, 24]. The international Kidney Disease: Improving Global Outcomes guidelines for CKD [4, 5], UK National Institute for Health and Care Excellence guidelines for HK [25–27], the HF guidelines from the American College of Cardiology/American Heart Association/Heart Failure Society of America [7], and the European Society of Cardiology (ESC) [6] all emphasize the importance of managing HK to facilitate RAASi therapy, and recommend using anti-HK treatment with potassium binders. For example, the ESC 2021 HF guidelines stipulate that in patients with HK who are not on maximally tolerated, guideline-recommended target dose of RAASi, an anti-HK agent may be initiated, RAASi therapy should be up-titrated with close monitoring of potassium levels, and upon achieving normokalemia, the anti-HK treatment should be maintained [6]. Considering the suboptimal tolerability profile of conventional binders, which hampers their long-term use, the guidelines recommend newer anti-HK agents (patiromer and sodium zirconium cyclosilicate [SZC]).

However, down-titration or discontinuation of RAASi treatment is a common strategy to manage HK in routine clinical practice, as opposed to targeted concomitant management with anti-HK treatment [16, 28, 29]. In an analysis of patients on a maximum dose of RAASi in a large US electronic medical records database, RAASi down-titration or discontinuation occurred among 38–47% of patients after an episode of HK [16].

Previous research has focused on the clinical impact of not achieving maximum RAASi dose, without necessarily accounting for the underlying rationale and that some patients may not tolerate the maximum dose due to adverse effects unrelated to HK. This study provides a direct comparison of the impact of maintained versus down-titrated or discontinued RAASi regimens (including, angiotensin-converting enzyme [ACE] inhibitors, angiotensin receptor blockers [ARBs], angiotensin receptor-neprilysin inhibitors [ARNis], and mineralocorticoid receptor antagonists [MRAs]) on cardiorenal risk following an episode of HK in patients with HF and/or CKD stage 3 or 4, in contemporary routine clinical practice in the US and Japan.

## Methods

### Data sources

This observational study included data from the US and Japan. Optum’s de-identified Market Clarity Data consists of electronic medical records linked with claims data via deterministic matching. The dataset includes information on patient demographics, date of death (year and month sourced from the Social Security Administration Death Master File), claims enrollment period, diagnoses, procedures and laboratory test results in inpatient and outpatient care, provider characteristics, issued and dispensed prescriptions, and medications administered in hospital. The Japan Medical Data Vision database captures claims data from hospitals across Japan, including information on diagnoses and procedures recorded in inpatient and outpatient hospital care, administration of in-hospital medications, and prescriptions. Laboratory test results are captured from a subset of hospitals.

### Study population

The study population included patients aged at least 18 years with an index episode of HK (International Classification of Diseases [ICD], Tenth Revision, E87.5 or ICD, Ninth Revision, 276.7) in any coding position between July 2019 and September 2021 (US), or May 2020 and September 2021 (Japan), with a history of HF or CKD stage 3 or 4 within the 6 months preceding the index episode (diagnosis codes are provided in Additional file 1), and at least one filled RAASi prescription within 6 months prior to index episode of HK.

Patients with a coded diagnosis of end-stage kidney disease (ESKD), eGFR < 15 ml/min/1.73 m^2^ or hemodialysis during baseline, or < 6 months of available data prior to index date were excluded. The index date was defined as the date of the episode of HK. In the case of > 1 recorded HK diagnosis, the index episode of HK was defined as the most recent HK diagnosis entered at least 6 months prior to the end of the data collection period in the respective data source (defined above), with the rationale to describe contemporary practice.

### Statistical analyses

Baseline patient characteristics, including demographics, comorbidities, and medication use, were described based on data from the 6 months prior to the index HK episode. Longitudinal patterns in RAASi treatment following the episode of HK were described as the percentage of patients attaining guideline-recommended target dose of RAASi (≤ 50, 51–75, and > 75%) in their most recent prescription within 90 days prior to index episode of HK, in their most recent prescription within 90 days after the index HK episode, and in their closest prescription prior to 6 months after index HK episode. Patients with missing dose data and those lost to follow-up before the end of the respective interval were excluded.

Patient-level changes in RAASi treatment following the HK episode were described according to the dose in the most recent prescription filled within 90 days after (and including) the index episode of HK relative to their most recent dose prior to (and excluding) the index HK episode. Patients were categorized as having maintained or up-titrated their dose, down-titrated their dose of any previously prescribed RAASi agent by > 25%, or discontinued (did not fill a new prescription within 90 days after index). Patients lost to follow-up prior to 90 days after the index HK episode were excluded to ensure all patients had equal possibility of filling a prescription within this time frame. Those with missing dose data were also excluded.

The cardiorenal composite outcome was defined as any of the following: hospitalization for HF (hospitalization with HF as main diagnosis), emergency visit for HF (diagnosis of HF in any position recorded in the emergency department or as the hospital admission diagnosis), and progression to ESKD (initiation of hemodialysis or a diagnosis of ESKD or CKD stage 5 in any position recorded in hospital, emergency, or outpatient setting). The risk of the cardiorenal composite outcome in relation to patient-level changes in RAASi treatment following an episode of HK was assessed at 6 months, using the Kaplan–Meier method. The components of the cardiorenal composite outcome were also analyzed separately for the CKD and HF patient cohorts. To avoid immortal time bias, the person-time at risk started on the day of the filled prescription in patients who filled a RAASi prescription within 90 days. In those without a filled prescription within 90 days, the person-time at risk started at the index HK episode. The risk of the cardiorenal composite outcome was described in relation to dose change for RAASi overall, as an average of all RAASi classes used by the patient, as well as per each RAASi class. Patients were censored at the end of continuous enrollment or last date of available data.

As death is a competing risk in the cardiorenal composite, the risk of the cardiorenal composite, including all-cause mortality, was also assessed. Also, the risk of all-cause mortality as an individual outcome was described.

To account for potential confounders in the association between patient-level changes in RAASi treatment and the risk of the cardiorenal composite outcome, a Cox proportional hazards regression model was applied. The adjusted model included age, sex, history of HK, diabetes, HF, CKD including stage, and baseline use of ACE inhibitors, ARBs, ARNis, and MRAs, respectively. Results were reported as hazard ratios (HRs) with 95% confidence interval (CI) and 2-sided *p*-values.

## Results

### Patient characteristics

Of the 15,488 patients from the US, 77% had CKD stage 3 or 4 (58% had stage 3 and 19% had stage 4), and 59% had HF (Table 1). In Japan (*N* = 6020), 24% of patients had CKD stage 3 or 4, and 89% had HF. Patients from Japan were older than those from the US, more often males, and had a higher level of sK^+^ at their index HK episode. The most common RAASi classes were ACE inhibitors in the US and ARBs in Japan.

**Table 1:** Patient characteristics at baseline

Characteristic	US	Japan
	***N*** = 15,488	***N*** = 6020
^a^Excluded from the denominator for non-missing data		
Age at index, years		
Mean (SD)	70.2 (12.4)	76.8 (11.4)
Median (IQR)	71 (62–80)	78 (71–85)
Male, *n* (%)	8176 (52.8)	3810 (63.3)
History of HK, *n* (%)	6064 (39.2)	961 (16.0)
HK severity at index, *n* (%)		
> 5.0–5.49	4940 (48.6)	207 (32.8)
5.5–5.99	3414 (33.6)	268 (42.5)
≥ 6	1805 (17.8)	156 (24.7)
Missing^a^	5329 (34.4)	5389 (89.5)
Diabetes, *n* (%)	10,236 (66.1)	2550 (42.4)
CKD stage by diagnosis code or by eGFR, *n* (%)		
3 or 4	11,873 (76.7)	1427 (23.7)
3	8931 (57.7)	534 (8.9)
4	2942 (19.0)	893 (14.8)
HF, *n* (%)	9086 (58.7)	5348 (88.8)
RAASi, *n* (%)		
ACEi	8886 (57.4)	1281 (21.3)
ARB	4786 (30.9)	4037 (67.1)
ARNi	758 (4.9)	38 (0.6)
MRA	3803 (24.6)	2168 (36.0)

*ACEi* Angiotensin-converting enzyme inhibitor, *ARB* Angiotensin receptor blocker, *ARNi* Angiotensin receptor-neprilysin inhibitor, *CKD* Chronic kidney disease, *eGFR* Estimated glomerular filtration rate, *HF* Heart failure, *HK* Hyperkalemia, *IQR* Interquartile range, *MRA* Mineralocorticoid receptor antagonist, *RAASi* Renin-angiotensin-aldosterone system inhibitor, *SD* Standard deviation

Characteristics of the subgroup with CKD stage 3 or 4 (irrespective of HF) and of the subgroup with HF (irrespective of CKD stage 3 or 4) are presented in Additional files 2 and 3, respectively.

### Longitudinal patterns of RAASi guideline-recommended target dose attainment

**Fig. 1:**
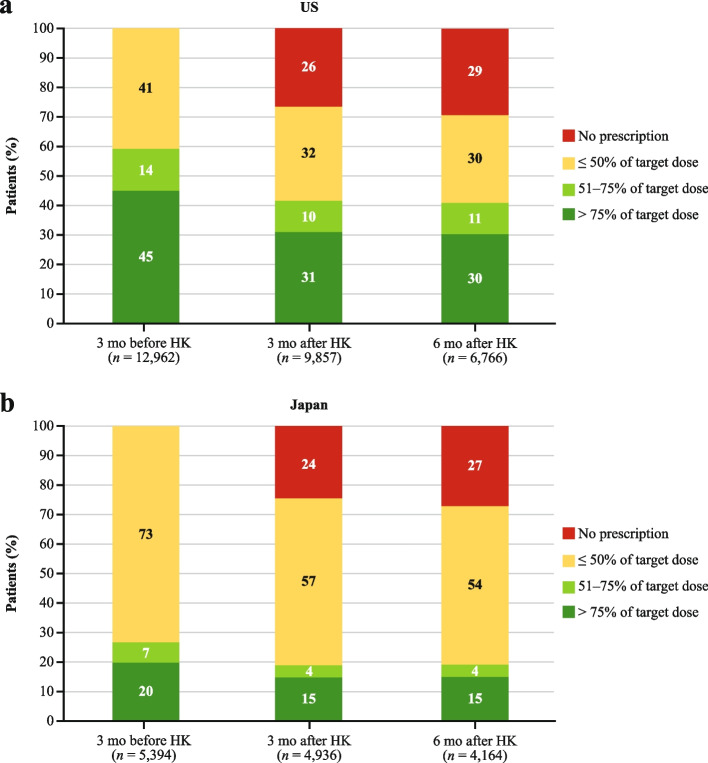
Attainment of guideline-recommended RAASi dose in (**a**) the US and (**b**) Japan. *HK* hyperkalemia, *mo* month, *RAASi* renin-angiotensin-aldosterone system inhibitor

RAASi dose attainment in relation to local guideline-recommended target dose was described over time among patients who filled a RAASi prescription in the 3 months prior to the episode of HK (*N* = 12,962 in the US and *N* = 5394 in Japan). In the US, 59% attained > 50% of the target dose across all RAASi classes prior to the HK episode (Fig. 1). In the subsequent 3–6 months, this dropped to 41%; 26–29% did not fill any new RAASi prescription. In Japan, target dose attainment prior to HK was low, with only 27% attaining > 50% of the target dose across all RAASi classes prior to the HK episode (Fig. 1). In the subsequent 3–6 months, this dropped further to 19%, with 24–27% not filling any new RAASi prescription.

### RAASi change following an HK episode

**Fig. 2:**
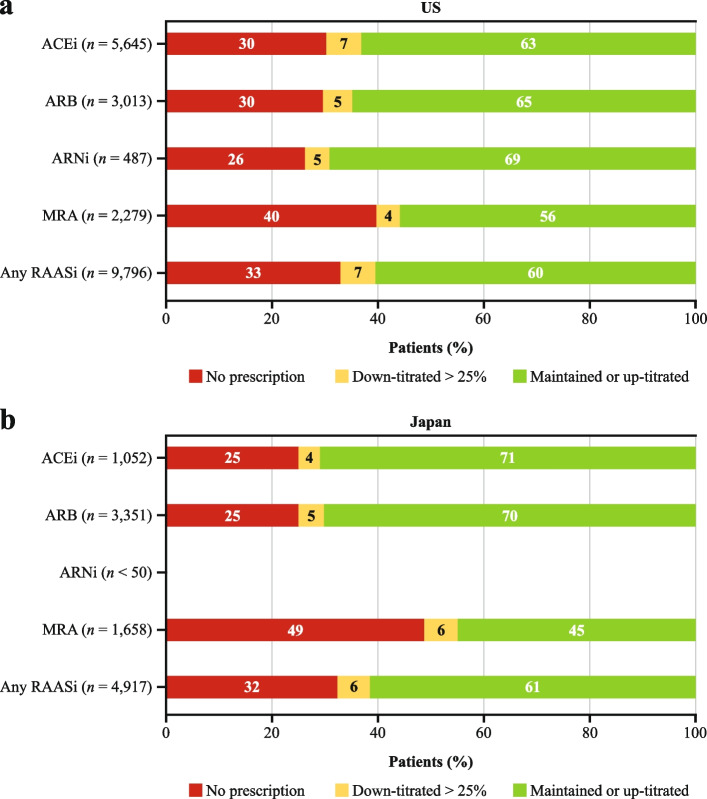
RAASi change following an HK episode in (**a**) the US and (**b**) Japan. *ACEi* angiotensin-converting enzyme inhibitor, *ARB* angiotensin receptor blocker, *ARNi* angiotensin receptor-neprilysin inhibitor, *HK* hyperkalemia, *MRA* mineralocorticoid receptor antagonist, *RAASi* renin-angiotensin-aldosterone system inhibitor

Figure 2 describes the percentage of patients who maintained or up-titrated their dose, down-titrated by > 25%, or did not fill a new prescription within 90 days after the HK episode. Overall, 33% (US) and 32% (Japan) of patients did not fill a new RAASi prescription, and 7% (US) and 6% (Japan) down-titrated at least one of their RAASi treatments by > 25% after the HK episode. One percent of patients in the US and Japan, respectively, filled a prescription for a newer anti-HK agent (SZC or patiromer) within 3 months of the HK episode.

The characteristics of those who maintained or up-titrated, discontinued, or down-titrated their RAASi treatment are presented in Table 2. In both the US and Japan, a higher proportion of patients who discontinued or down-titrated experienced a more severe index HK episode (sK^+^ ≥ 6.0 mmol/L) compared with those who maintained or up-titrated. Baseline RAASi use per class was similar, except for more frequent use of MRAs in those who discontinued or down-titrated.

**Table 2:** Patient characteristics by change in RAASi dose following an HK episode

	**US**			**Japan**		
	**Maintained or up-titrated**	**Discontinued**	**Down-titrated**	**Maintained or up-titrated**	**Discontinued**	**Down-titrated**
**Characteristic**	***N*** **= 5922**	***N*** **= 3223**	***N*** **= 650**	***N*** **= 3023**	***N*** **= 1591**	***N*** **= 303**
^a^Excluded from the denominator for non-missing data						
Age at index, years						
Mean (SD)	69.5 (12.3)	70.6 (12.4)	68.7 (12.3)	75.5 (11.6)	78.1 (11.2)	76.6 (11.1)
Median (IQR)	70 (61–79)	72 (62–80)	69 (60–78)	77 (70–84)	79.0 (73–86)	78 (72–85)
Male, *n* (%)	3135 (52.9)	1669 (51.8)	343 (52.8)	1977 (65.4)	950 (59.7)	199 (65.7)
History of HK, *n* (%)	2137 (36.1)	1229 (38.1)	224 (34.5)	473 (15.6)	257 (16.2)	42 (13.9)
HK severity at index, *n* (%)						
> 5.0–5.49	1941 (51.9)	956 (44.0)	212 (47.3)	118 (34.6)	32 (25.8)	< 11 pts
5.5–5.99	1239 (33.1)	757 (34.8)	138 (30.8)	166 (48.7)	42 (33.9)	14 (56.0)
≥ 6	561 (15.0)	462 (21.2)	98 (21.9)	57 (16.7)	50 (40.3)	< 11 pts
Missing^a^	2181 (36.8)	1048 (32.5)	202 (31.1)	2682 (88.7)	1467 (92.2)	278 (91.7)
Diabetes at baseline, *n* (%)	3848 (65.0)	2137 (66.3)	441 (67.8)	1245 (41.2)	699 (43.9)	132 (43.6)
CKD stage at baseline by diagnosis code or by eGFR, *n* (%)						
3 or 4	4586 (77.4)	2460 (76.3)	460 (70.8)	793 (26.2)	323 (20.3)	63 (20.8)
3	3601 (60.8)	1829 (56.7)	357 (54.9)	299 (9.9)	115 (7.2)	20 (6.6)
4	985 (16.6)	631 (19.6)	103 (15.8)	494 (16.3)	208 (13.1)	43 (14.2)
HF at baseline, *n* (%)	3048 (51.5)	1966 (61.0)	430 (66.2)	2617 (86.6)	1460 (91.8)	278 (91.7)
RAASi at baseline, *n* (%)						
ACEi	3475 (58.7)	1922 (59.6)	413 (63.5)	658 (21.8)	356 (22.4)	72 (23.8)
ARB	1895 (32.0)	1051 (32.6)	198 (30.5)	2167 (71.7)	1021 (64.2)	199 (65.7)
ARNi	283 (4.8)	190 (5.9)	43 (6.6)	18 (0.6)	14 (0.9)	< 11 pts
MRA	1208 (20.4)	1062 (33.0)	210 (32.3)	730 (24.1)	823 (51.7)	169 (55.8)

*ACEi* Angiotensin-converting enzyme inhibitor, *ARB* Angiotensin receptor blocker, *ARNi* Angiotensin receptor-neprilysin inhibitor, *CKD* Chronic kidney disease, *eGFR* Estimated glomerular filtration rate, *HF* Heart failure, *HK* Hyperkalemia, *IQR* Interquartile range, *MRA* Mineralocorticoid receptor antagonist, *pts *Patients, *RAASi* Renin-angiotensin-aldosterone system inhibitor, *SD* Standard deviation

### Risk of cardiorenal outcomes by change in RAASi following an HK episode

**Fig. 3:**
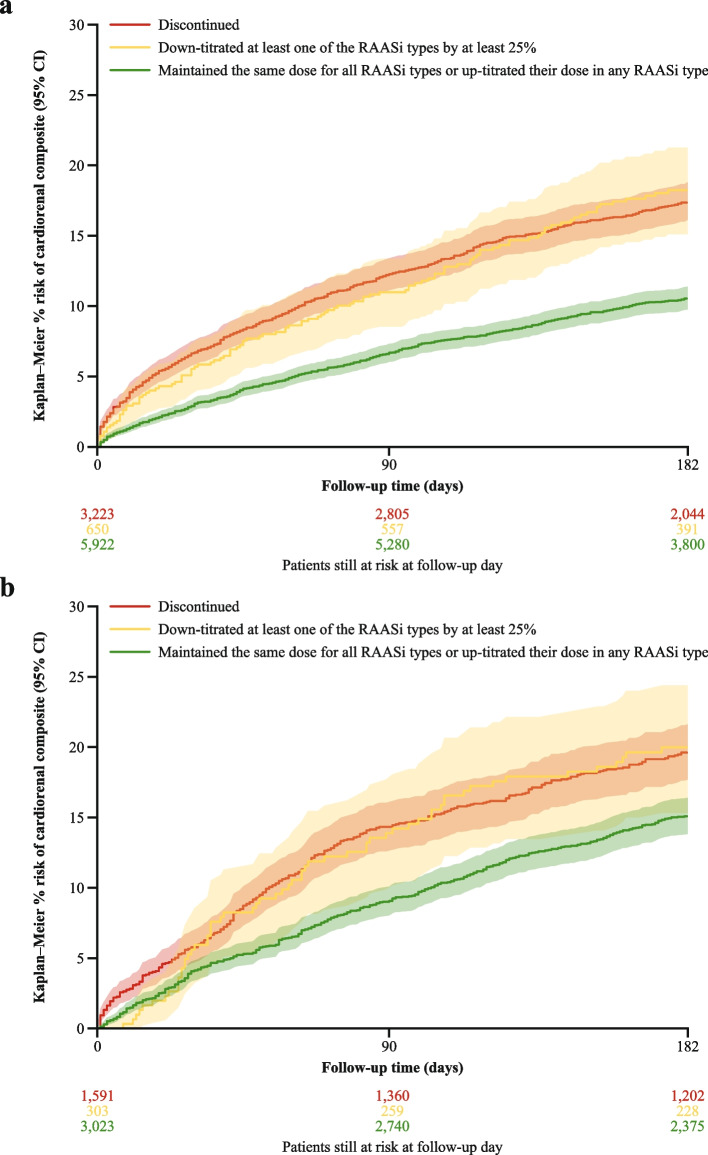
Risk of cardiorenal composite outcome by change in RAASi treatment following HK episode: (**a**) the US, (**b**) Japan. *CI* confidence interval, *HK* hyperkalemia, *RAASi* renin-angiotensin-aldosterone system inhibitor. *P*-value for the differences between the groups: US, *p* < 0.001; Japan, *p* < 0.001

The risk of the cardiorenal composite outcome was considerably higher in patients who discontinued or down-titrated versus maintained or up-titrated a RAASi treatment (Fig. 3). In the US, the risk at 6 months was 17.5% (95% CI 16.1–18.8%) in those who discontinued, 18.2% (15.1–21.3%) in those who down-titrated, and 10.6% (9.8–11.4%) in those who maintained or up-titrated (*p* <  0.001). In Japan, the corresponding risk was 19.7% (17.7–21.6%) in those who discontinued, 20.0% (15.3–24.4%) in those who down-titrated, and 15.1% (13.8–16.4%) in those who maintained or up-titrated (*p* <  0.001).

The number of patients who down-titrated their RAASi treatment was small, but results suggest that their risk is higher than in those who maintained or up-titrated.

While some categories included small numbers of patients, the trend of an increased risk of the cardiorenal composite outcome associated with discontinuation of RAASi was also observed in each of the individual RAASi classes (Table 3).

**Table 3:** Six-month risk of cardiorenal composite outcome by change in each RAASi class following an HK episode

RAASi class	US			Japan		
	***N***	6-month risk, % (95% CI)	***p-***value^**a**^	***N***	6-month risk, % (95% CI)	***p-***value^**a**^
ACEi						
Maintained or up-titrated	3564	9.6 (8.6–10.6)		745	19.4 (16.5–22.2)	
Down-titrated (> 25%)	373	17.1 (13.0–21.0)		44	29.5 (14.7–41.8)	
Discontinued	1708	16.3 (14.5–18.1)	< 0.001	263	21.8 (16.6–26.6)	0.37
ARB						
Maintained or up-titrated	1954	10.2 (8.8–11.5)		2350	14.1 (12.7–15.6)	
Down-titrated (> 25%)	163	20.5 (13.7–26.7)		159	19.0 (12.6–24.9)	
Discontinued	896	20.6 (17.9–23.2)	< 0.001	842	19.9 (17.2–22.6)	< 0.001
ARNi						
Maintained or up-titrated	337	20.2 (15.7–24.5)		26	< 11 pts	
Down-titrated (> 25%)	22	< 11 pts		< 11 pts	< 11 pts	
Discontinued	128	34.4 (25.4–42.2)	< 0.001	< 11 pts	< 11 pts	N/A
MRA						
Maintained or up-titrated	1271	18.1 (15.8–20.2)		744	17.7 (14.9–20.4)	
Down-titrated (> 25%)	100	17.9 (9.7–25.2)		106	16.3 (8.9–23.2)	
Discontinued	908	20.9 (18.1–23.5)	0.14	808	22.1 (19.2–25.0)	0.02

*ACEi* Angiotensin-converting enzyme inhibitor, *ARB* Angiotensin receptor blocker, *ARNi* Angiotensin receptor-neprilysin inhibitor, *CI* Confidence interval, *HK* Hyperkalemia, *MRA* Mineralocorticoid receptor antagonist, *N/A* Not applicable, *pts* Patients, *RAASi* Renin-angiotensin-aldosterone system inhibitor, *SD* Standard deviation

^a^Maintained or up-titrated versus discontinued

Following adjustment for potential confounders, the risk of the cardiorenal composite remained higher in those who discontinued than in those who maintained their RAASi treatment, in both the US (HR 1.55, 95% CI 1.38–1.75, *p* <  0.001) and Japanese cohorts (HR 1.24, 95% CI 1.07–1.44, *p* = 0.005) (Table 4).

**Table 4:** Risk of the cardiorenal composite adjusted for potential confounders

	Unadjusted		Adjusted^a^	
	HR (95% CI)^b^	***p***-value	HR (95% CI)^b^	***p***-value
**US**				
Discontinued	1.75 (1.56–1.97)	< 0.001	1.55 (1.38–1.75)	< 0.001
Down-titrated	1.80 (1.47–2.20)	< 0.001	1.51 (1.24–1.86)	< 0.001
**Japan**				
Discontinued	1.36 (1.18–1.57)	< 0.001	1.24 (1.07–1.44)	0.005
Down-titrated	1.37 (1.05–1.80)	0.021	1.25 (0.95–1.65)	0.105

*CI* Confidence interval, *HR* Hazard ratio

^a^Adjusted for age, sex, history of hyperkalemia, diabetes, heart failure, chronic kidney disease including stage, and baseline use of angiotensin-converting enzyme inhibitors; angiotensin receptor blockers, angiotensin receptor-neprilysin inhibitors, and mineralocorticoid receptor antagonists, respectively

^b^Risk relative to maintained renin-angiotensin-aldosterone system inhibitor treatment

The risk patterns remained consistent when adding mortality into the cardiorenal composite. In the US, the risk at 6 months was 20.5% (95% CI 19.1–22.0%) in those who discontinued their RAASi treatment versus 13.4% (12.5–14.3%) in those who maintained or up-titrated. In Japan, the corresponding risks were 21.8% (19.7–23.8%) and 16.3% (15.0–17.6%).

In the US subgroup with CKD stage 3 or 4 (irrespective of HF), the risk of the cardiorenal composite was 17.3% (95% CI 15.7–18.8%) in those who discontinued and 10.0% (9.1–10.9%) in those who maintained or up-titrated (Additional file 4). In Japan, the corresponding risks were 17.5% (13.2–21.5%) and 12.8% (10.4–15.1%).

In the US subgroup with HF (irrespective of CKD), the risk of the cardiorenal composite was 24.5% (95% CI 22.5–26.4%) in those who discontinued and 16.6% (15.2–18.0%) in those who maintained or up-titrated (Additional file 5). In Japan, the corresponding risks were 20.8% (18.6–22.8%) and 16.3% (14.8–17.7%).

The 6-month risks of the components of the cardiorenal composite are presented in Table 5, and with Kaplan–Meier plots in Additional file 6. Overall, the pattern of a lower risk of cardiorenal events with maintained or up-titrated RAASi treatment was also observed for the individual components of the cardiorenal composite outcome. As mentioned above, the number of patients with down-titration was low.

**Table 5:** Six-month progression risk to ESKD and HF composite by change in RAASi dose following an HK episode

RAASi change	Event	US			Japan		
		6-month risk, % (95% CI)	Patient count	Events (***N***)	6-month risk, % (95% CI)	Patient count	Events (***N***)
Maintained or up-titrated	Progression to ESKD^a^ in patients with CKD stage 3 or 4	3.2 (2.7–3.8)	4586	138	5.3 (3.7–6.9)	793	41
Down-titrated		5.4 (3.2–7.5)	460	23	–	63	–
No prescription		5.8 (4.9–6.8)	2460	138	8.1 (5.1–11.0)	323	26
Maintained or up-titrated	HF composite^b^ in patients with HF	14.6 (13.3–15.9)	3049	421	11.7 (10.5–13.0)	2617	304
Down-titrated		20.8 (16.7–24.7)	430	84	18.9 (14.1–23.4)	278	52
No prescription		20.6 (18.7–22.4)	1966	392	16.2 (14.2–18.0)	1460	234

*CI* Confidence interval, *CKD* Chronic kidney disease, *ESKD* End-stage kidney disease, *HF* Heart failure, *HK* Hyperkalemia, *RAASi* Renin-angiotensin-aldosterone system inhibitor

Events (*N*), number of events at 6 months

^a^Including diagnosis of CKD stage 5/ESKD or initiation of hemodialysis

^b^Hospitalization for HF, emergency visit for HF

In the US, the 6-month risk of all-cause mortality was 5.3% (95% CI 4.5–6.1%) in those who discontinued and 3.8% (3.3–4.3%) in those who maintained or up-titrated. In Japan, the corresponding risks were 4.1% (3.2–5.1%) and 2.4% (1.8–2.9%).

## Discussion

RAASi treatment is a cornerstone in the management of patients with CKD and/or HF [1–3]. This study assessed the impact of an HK-related reduction in RAASi therapy from a cardiorenal perspective. Despite guideline recommendations to maintain RAASi therapy with newer anti-HK treatment, RAASi therapy in contemporary clinical practice is commonly discontinued following an HK episode, and the RAASi reduction often persists over time.

The outcome assessed in this analysis was a cardiorenal composite, including HF hospitalization or emergency visit, or progression to CKD stage 5, assessed in a population with HF and/or CKD stage 3 or 4. From the comparisons of the impact of maintained versus either down-titrated or discontinued RAASi regimens on cardiorenal risk, the findings showed that HK-related RAASi down-titration or discontinuation are each associated with a comparable, but higher, risk of subsequent cardiorenal events compared with maintained or up-titrated RAASi. Notably, a large observational study based on 12-month follow-up data showed that the incidence of the composite measure of any adverse outcome or mortality in patients on maximum doses was comparable with those on submaximal doses (each 24.9%), and lower than for patients who discontinued RAASi (~ 34%); for mortality alone, these rates were 4.1, 8.2, and 11.0%, respectively [16]. More recently, an observational cohort study in Canada similarly found that RAASi discontinuation was associated with a higher risk of mortality and cardiovascular events compared with continuation [30]. In the current study, the characteristics of patients who maintained or up-titrated versus down-titrated or discontinued their RAASi treatment were similar, except that patients who discontinued had a more severe index HK episode (sK^+^ ≥ 6.0 mmol/L) than those who maintained or up-titrated, and a higher use of MRA at baseline. Still, the association of a higher risk of the cardiorenal composite in those who discontinued versus maintained their RAASi treatment remained after adjustment for potential confounders, including baseline MRA use.

In clinical practice, MRAs have tended to be reserved for patients with more severe HF [31], although recent HF guidelines now recommend use of an MRA in all patients with HF with reduced left ventricular ejection fraction (regardless of severity) [6, 7]. Our findings showed that the risk of the cardiorenal composite outcome was somewhat higher in patients receiving MRA than those receiving ACE inhibitors or an ARB. However, it should be noted that the observed higher proportion of patients who were receiving an MRA in those who discontinued RAASi may have been a confounder, given that MRA use is likely a marker for HF severity. However, the pattern of an increased risk associated with RAASi discontinuation was observed across all individual RAASi classes; even in patients with baseline MRA, the results indicate that maintained MRA treatment tended to be associated with a lower risk of cardiorenal events compared with discontinuation.

The differences between the populations, healthcare settings, and recommended target doses of RAASi in the US and Japan means it is not possible to directly compare the outcomes between the two countries. One difference was the distribution of HF and CKD. The US population had a higher proportion of patients with CKD stage 3 and stage 4 (77 vs. 24% in Japan), while the occurrence of HF was relatively higher in Japan (89 vs. 59% in the US). Furthermore, CKD stage 3, as determined via a diagnostic code with staging details or via eGFR values, was recorded among 58% of the US population, corresponding to 75% of those with CKD stage 3 or 4. However, in Japan, CKD stage 3 diagnosis rate was comparatively low, being 9% in total (37% of those with CKD stage 3 or 4); a potential consideration for data interpretation. Target RAASi dose attainment prior to the HK episode in Japan was particularly low (27% attaining > 50% of the target dose across all RAASi classes); coupled with further reduction to 19% at 6 months post-HK and nearly 30% discontinuing RAASi, highlights a large unmet need in Japan for target dose attainment and need for strategies to increase guideline awareness.

The strengths of the analysis include the large populations studied from the US and Japan, and the rich data obtained from the respective databases. However, the analysis is limited to the data captured in the databases, and information on race, certain risk factors, and severity markers for HF and CKD were not available.

Since the rationale of the study was to describe the cardiorenal consequences of HK-related reductions in RAASi therapy, the exposure (change in RAASi treatment) was determined over a short follow-up window and in close connection with the HK episode, i.e., within 90 days. It is possible that patients who had their RAASi treatment discontinued or down-titrated in connection with the HK episode may have resumed or up-titrated their RAASi treatment at some point later during the 6-month outcomes assessment period. Clinically, it is important that patients are carefully monitored following an HK event so that clinicians can determine when to restart RAASi treatment. While the analyses on longitudinal patterns of RAASi treatment were assessed cross-sectionally, the results suggest that treatment changes made following an episode of HK tend to persist over the following 6 months, similarly in culturally diverse populations, strengthening the validity of the findings. Previous research in a UK cohort also shows that once RAASi is discontinued or down-titrated following an HK episode, it is often not restarted or up-titrated [22, 24].

Another potential limitation of this analysis is that the starting point for the outcomes assessment differed between exposure categories (first filled prescription within 90 days vs. HK episode in those with no prescription). In alignment with the main goal of the study, i.e., to assess the cardiorenal risk associated with HK-related reduction in RAASi treatment, it was crucial to ensure an equal possibility of capturing a filled prescription in all exposure categories. All patients were therefore required to be alive and with available follow-up at 90 days. However, this requirement also leads to the exclusion of the sickest population who die within the first 90 days, and underestimates the risk of death within the first 90 days. This may also have slightly underestimated the risk of the main cardiorenal outcomes, such as hospitalization for HF, in case patients have such an event and die within the first 90 days.

The analyses of the main outcome did not account for the competing risk of death. To address the potential impact of death as a competing risk, additional analyses were performed by adding mortality into the composite. The resulting risk patterns remained consistent with the main analyses.

The patients in this analysis were identified in 2019–2021 (US) and 2020–2021 (Japan), so precede the release of the new HF guidelines [6, 7]. Furthermore, the dataset does not include patients who received the non-steroidal selective MRA finerenone, which was approved for patients with CKD associated with type 2 diabetes in the US in July 2021 [32] and in Japan in March 2022 [33]. In the FIDELIO-DKD clinical trial, the incidence of HK appeared to be lower with finerenone than with steroidal MRAs, such as spironolactone, although it was still associated with a 2-fold higher risk of HK than placebo [34]. Future studies are needed to investigate the risk of HK with finerenone in routine clinical practice. Additional studies should include evaluation of the factors that influence clinician RAASi prescribing practice following an HK episode, including the impact of location/geography, healthcare setting, and specialty.

## Conclusion

RAASi discontinuation or down-titration after an episode of HK was associated with a higher risk of cardiorenal events compared with maintained or up-titrated RAASi. These data emphasize the importance of RAASi optimization following an episode of HK and the need for targeted treatment of HK to facilitate optimal RAASi management.

## Supplementary Information

**Additional file 1.**

**Additional file 2.** Patient characteristics at baseline in patients with CKD stage 3 or 4 (with or without HF).

**Additional file 3.** Patient characteristics at baseline in patients with HF (with or without CKD stage 3 or 4).

**Additional file 4.** Risk of the cardiorenal composite outcome by change in RAASi dose following an HK episode in (a) the US and (b) Japan in patients with CKD stage 3 or 4 (with or without HF).

**Additional file 5.** Risk of the cardiorenal composite outcome by change in RAASi dose following an HK episode in (a) the US and (b) Japan in patients with HF (with or without CKD stage 3 or 4).

**Additional file 6.** Risk of ESKD progression in patients with CKD stage 3 or 4 (with or without HF) in (a) the US and (b) Japan, and risk of the HF composite in patients with HF (with or without CKD stage 3 or 4) in (c) the US and (d) Japan.

## Data Availability

Data underlying the findings described in this manuscript may be obtained via the corresponding author upon reasonable request, in accordance with AstraZeneca’s data sharing policy described at link: https://astrazenecagrouptrials.pharmacm.com/ST/Submission/Disclosure. However, restrictions apply to these data, which were used under license for the current study and are not publicly available.

## Abbreviations

ACEi: Angiotensin-converting enzyme inhibitor
ARB: Angiotensin receptor blocker
ARNi: Angiotensin receptor-neprilysin
CI: Confidence interval
CKD: Chronic kidney disease
eGFR: Estimated glomerular filtration rate
ESC: European Society of Cardiology
ESKD: End-stage kidney disease
HF: Heart failure
HK: Hyperkalemia
HR: Hazard ratio
ICD: International Classification of Diseases
IQR: Interquartile range
MRA: Mineralocorticoid receptor antagonist
RAASi: Renin-angiotensin-aldosterone system inhibitor
SD: Standard deviation
sK^+^: Serum potassium
SZC: Sodium zirconium cyclosilicate
