# Supplementary material for: Clinical impact of suboptimal RAASi therapy following an episode of hyperkalemia
Source: BMC Nephrol. 2023 Jan 19;24:18. doi: 10.1186/s12882-022-03054-5 (PMC9854063; doi:10.1186/s12882-022-03054-5)
Supplement: Supplementary file 2 — Additional file 2. Patient characteristics at baseline in patients with CKD stage 3 or 4 (with or without HF). [file 12882_2022_3054_MOESM2_ESM.docx]

Additional File 2 Patient characteristics at baseline in patients with CKD stage 3 or 4 (with or without HF)

|  | **US** | **Japan** |
| --- | --- | --- |
| **Characteristic** | ***N* = 11,873** | ***N* = 1,427** |
| ^a^Excluded from the denominator for non-missing data | | |
| Age at index, years |  |  |
| Mean (SD) | 71.2 (12.1) | 76.3 (11.5) |
| Median (IQR) | 73 (63–81) | 78 (71–84) |
| Male, *n* (%) | 6,168 (51.9) | 938 (65.7) |
| HK diagnosis, *n* (%) | 4,981 (42.0) | 223 (15.6) |
| HK severity at index, *n* (%) |  |  |
| > 5.0–5.49 | 3,912 (49.1) | 157 (31.2) |
| 5.5–5.99 | 2,683 (33.7) | 222 (44.0) |
| ≥ 6 | 1,375 (17.3) | 125 (24.8) |
| Missing^a^ | 3,903 (32.9) | 923 (64.7) |
| Diabetes, *n* (%) | 8,132 (68.5) | 618 (43.3) |
| CKD stage by diagnosis code or by eGFR, *n* (%) |  |  |
| CKD (stage 3 or 4) | 11,873 (100.0) | 1,427 (100.0) |
| CKD stage 3 | 8,931 (75.2) | 534 (37.4) |
| CKD stage 4 | 2,942 (24.8) | 893 (62.6) |
| HF, *n* (%) | 5,471 (46.1) | 755 (52.9) |
| RAASi, *n* (%) |  |  |
| ACEi | 6,734 (56.7) | 227 (15.9) |
| ARB | 3,828 (32.2) | 1,103 (77.3) |
| ARNi | 453 (3.8) | < 11 patients |
| MRA | 2,646 (22.3) | 386 (27.0) |

*ACEi* angiotensin-converting enzyme inhibitor, *ARB* angiotensin receptor blocker, *ARNi* angiotensin receptor-neprilysin inhibitor, *CKD* chronic kidney disease, *eGFR* estimated glomerular filtration rate, *HF* heart failure, *HK* hyperkalemia, *IQR* interquartile range, *MRA* mineralocorticoid receptor antagonist, *RAASi* renin-angiotensin-aldosterone system inhibitor, *SD* standard deviation.
