# Supplementary material for: Clinical impact of suboptimal RAASi therapy following an episode of hyperkalemia
Source: BMC Nephrol. 2023 Jan 19;24:18. doi: 10.1186/s12882-022-03054-5 (PMC9854063; doi:10.1186/s12882-022-03054-5)
Supplement: Supplementary file 3 — Additional file 3. Patient characteristics at baseline in patients with HF (with or without CKD stage 3 or 4). [file 12882_2022_3054_MOESM3_ESM.docx]

Additional File 3 Patient characteristics at baseline in patients with HF (with or without CKD stage 3 or 4)

|  | **US** | **Japan** |
| --- | --- | --- |
| **Characteristic** | ***N* = 9,086** | ***N* = 5,348** |
| ^a^Excluded from the denominator for non-missing data | | |
| Age at index, years |  |  |
| Mean (SD) | 70.3 (12.5) | 77.1 (11.4) |
| Median (IQR) | 71 (62–81) | 79 (72–85) |
| Male, *n* (%) | 4,827 (53.1) | 3,351 (62.7) |
| HK diagnosis, *n* (%) | 3,443 (37.9) | 870 (16.3) |
| HK severity at index, *n* (%) |  |  |
| > 5.0–5.49 | 2,850 (48.6) | 124 (36.5) |
| 5.5–5.99 | 1,891 (32.3) | 142 (41.8) |
| ≥ 6 | 1,120 (19.1) | 74 (21.8) |
| Missing^a^ | 3,225 (35.5) | 5,008 (93.6) |
| Diabetes, *n* (%) | 6,009 (66.1) | 2,325 (43.5) |
| CKD stage by diagnosis code or by eGFR, *n* (%) |  |  |
| CKD (stage 3 or 4) | 5,471 (60.2) | 755 (14.1) |
| CKD stage 3 | 3,967 (43.7) | 272 (5.1) |
| CKD stage 4 | 1,504 (16.6) | 483 (9.0) |
| HF, *n* (%) | 9,086 (100.0) | 5,348 (100.0) |
| RAASi, *n* (%) |  |  |
| ACEi | 4,987 (54.9) | 3,470 (64.9) |
| ARB | 2,639 (29.0) | 38 (0.7) |
| ARNi | 747 (8.2) | 2,048 (38.3) |
| MRA | 2,988 (32.9) | 1,056 (19.7) |

*ACEi* angiotensin-converting enzyme inhibitor, *ARB* angiotensin receptor blocker, *ARNi* angiotensin receptor-neprilysin inhibitor, *CKD* chronic kidney disease, *eGFR* estimated glomerular filtration rate, *HF* heart failure, *HK* hyperkalemia, *IQR* interquartile range, *MRA* mineralocorticoid receptor antagonist, *RAASi* renin-angiotensin-aldosterone system inhibitor, *SD* standard deviation.
