# Supplementary material for: Clinical impact of suboptimal RAASi therapy following an episode of hyperkalemia
Source: BMC Nephrol. 2023 Jan 19;24:18. doi: 10.1186/s12882-022-03054-5 (PMC9854063; doi:10.1186/s12882-022-03054-5)
Supplement: Supplementary file 4 — Additional file 4. Risk of the cardiorenal composite outcome by change in RAASi dose following an HK episode in (a) the US and (b) Japan in patients with CKD stage 3 or 4 (with or without HF). [file 12882_2022_3054_MOESM4_ESM.docx]

Additional File 4 Risk of the cardiorenal composite outcome by change in RAASi dose following an HK episode in (a) the US and (b) Japan in patients with CKD stage 3 or 4 (with or without HF)


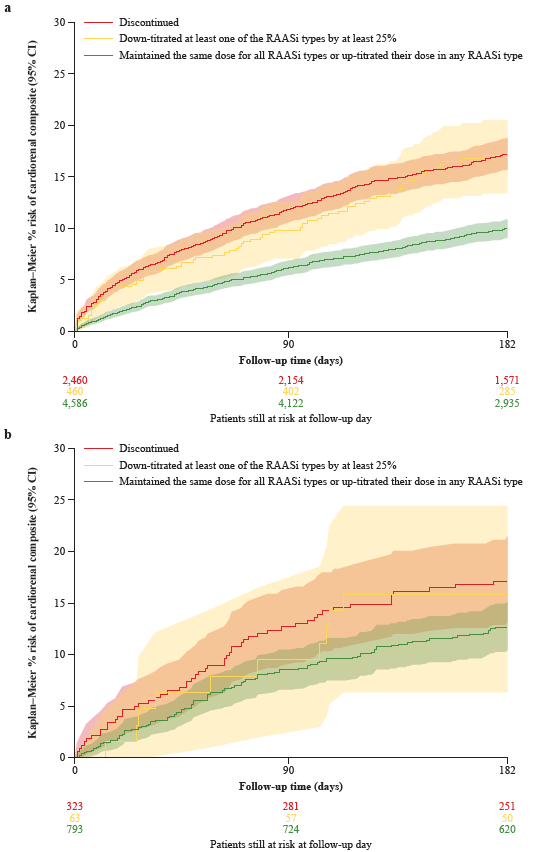


*CI* confidence interval, *CKD* chronic kidney disease, *HK* hyperkalemia, *RAASi* renin-angiotensin-aldosterone system inhibitor.
