# Supplementary material for: Clinical impact of suboptimal RAASi therapy following an episode of hyperkalemia
Source: BMC Nephrol. 2023 Jan 19;24:18. doi: 10.1186/s12882-022-03054-5 (PMC9854063; doi:10.1186/s12882-022-03054-5)
Supplement: Supplementary file 5 — Additional file 5. Risk of the cardiorenal composite outcome by change in RAASi dose following an HK episode in (a) the US and (b) Japan in patients with HF (with or without CKD stage 3 or 4). [file 12882_2022_3054_MOESM5_ESM.docx]

Additional File 5 Risk of the cardiorenal composite outcome by change in RAASi dose following an HK episode in (a) the US and (b) Japan in patients with HF (with or without CKD stage 3 or 4)


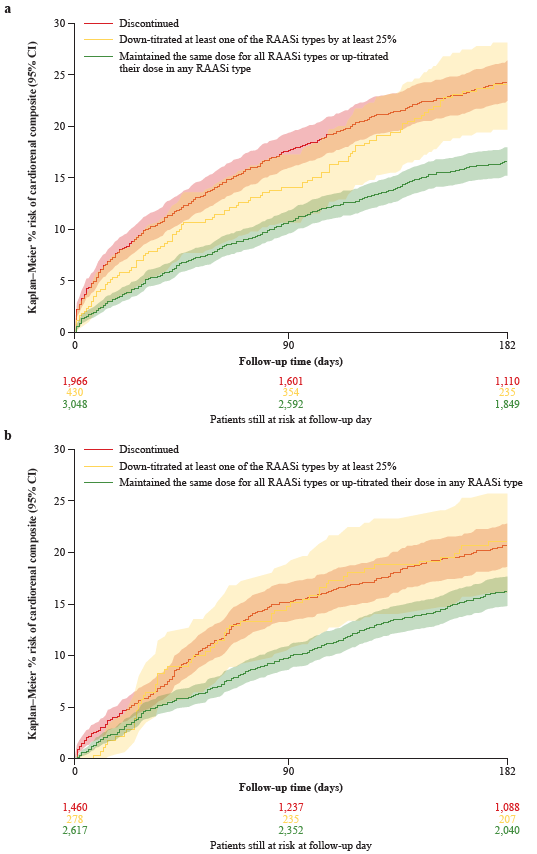


*CI* confidence interval, *CKD* chronic kidney disease, *HF* heart failure, *HK* hyperkalemia, *RAASi* renin-angiotensin-aldosterone system inhibitor.
