# Supplementary material for: Clinical impact of suboptimal RAASi therapy following an episode of hyperkalemia
Source: BMC Nephrol. 2023 Jan 19;24:18. doi: 10.1186/s12882-022-03054-5 (PMC9854063; doi:10.1186/s12882-022-03054-5)
Supplement: Supplementary file 6 — Additional file 6. Risk of ESKD progression in patients with CKD stage 3 or 4 (with or without HF) in (a) the US and (b) Japan, and risk of the HF composite in patients with HF (with or without CKD stage 3 or 4) in (c) the US and (d) Japan. [file 12882_2022_3054_MOESM6_ESM.docx]

Additional File 6 Risk of ESKD progression in patients with CKD stage 3 or 4 (with or without HF) in (a) the US and (b) Japan, and risk of the HF composite in patients with HF (with or without CKD stage 3 or 4) in (c) the US and (d) Japan

**
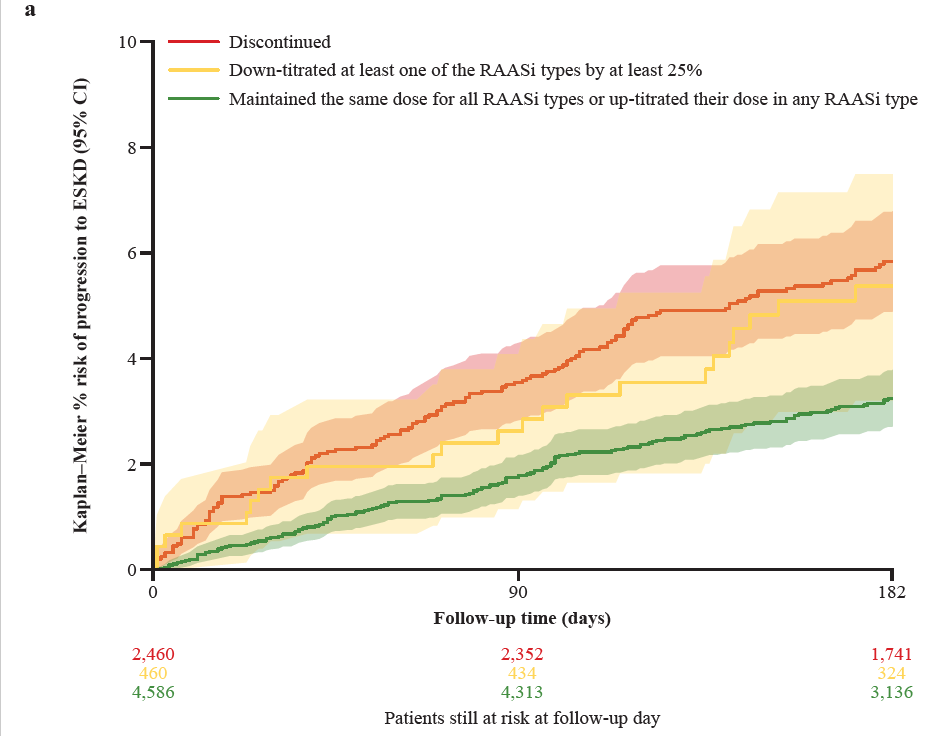
**

**
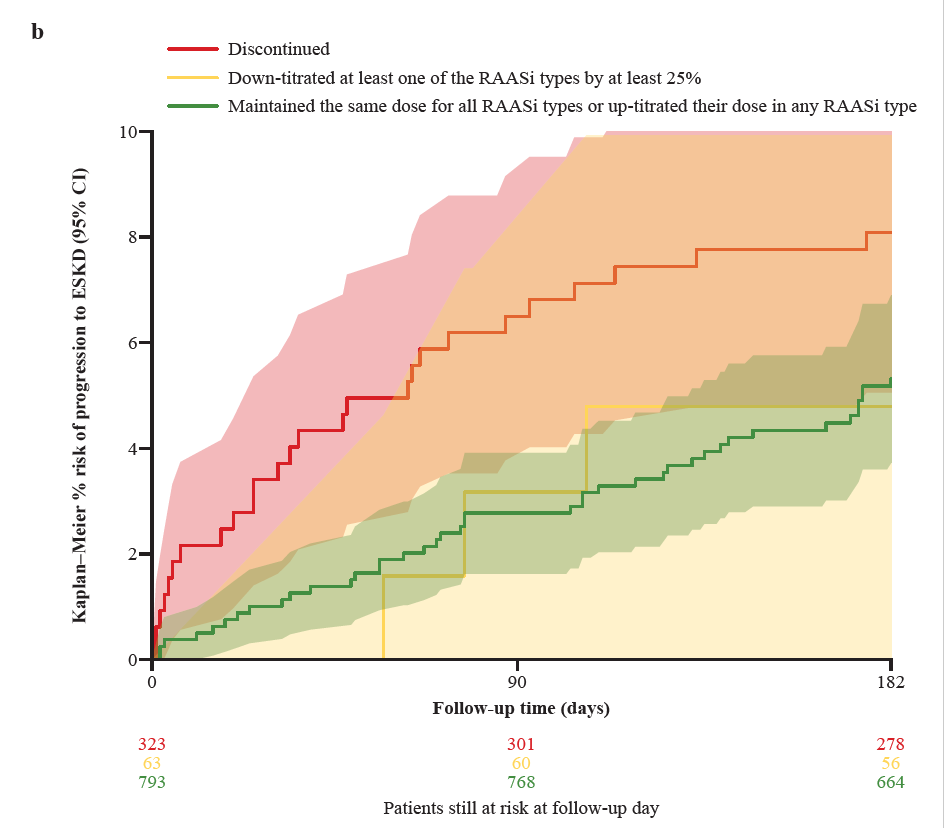
**

**
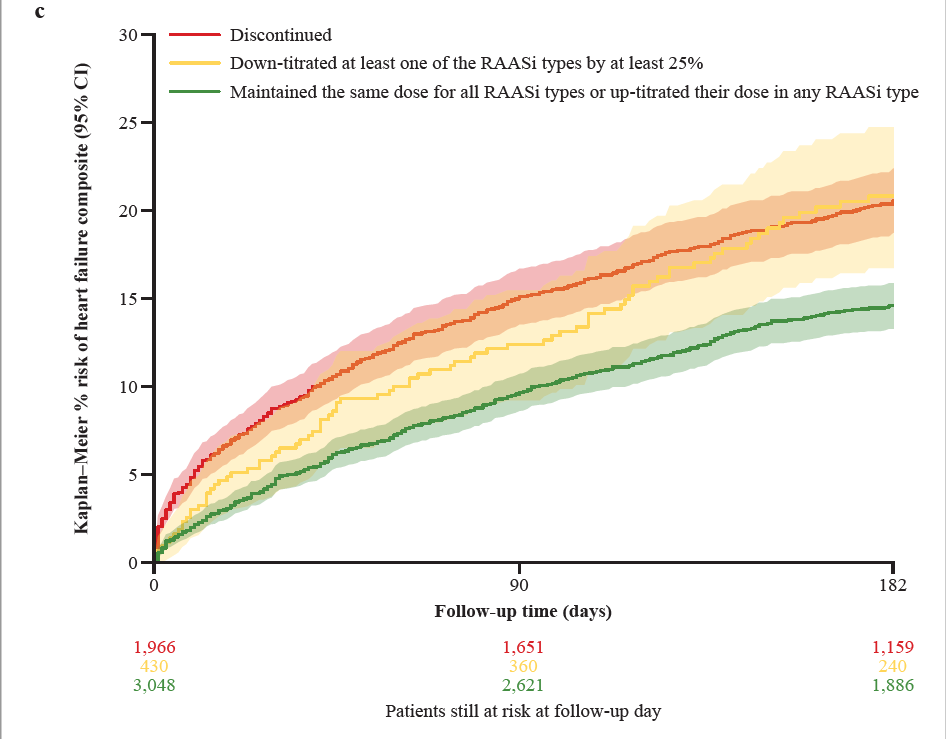
**

**
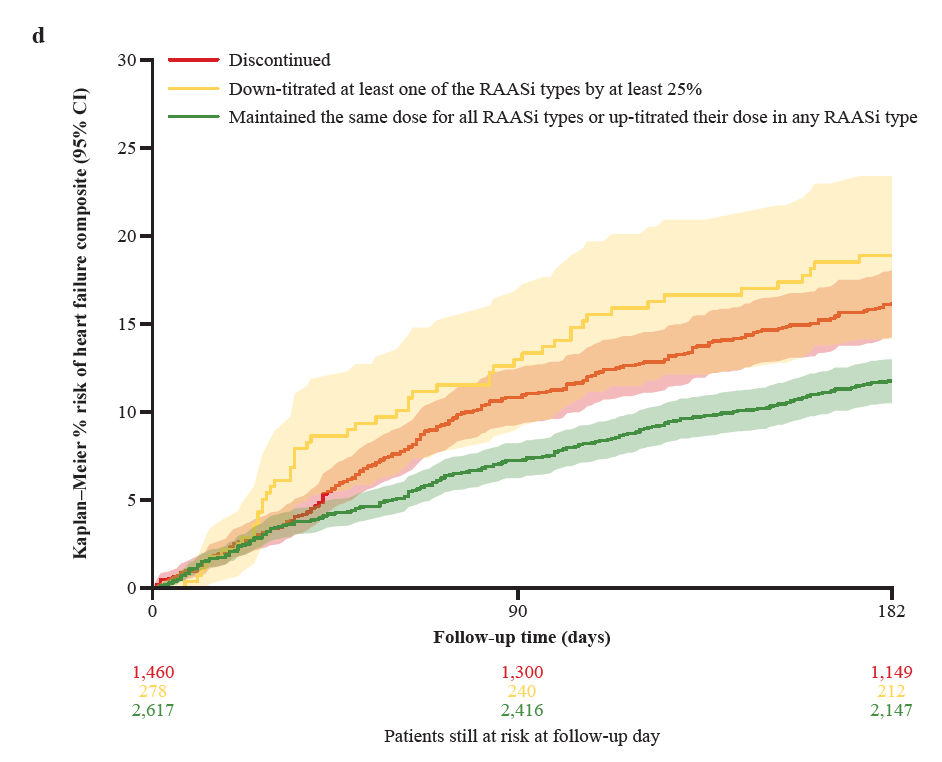
**

*CI* confidence interval, *CKD* chronic kidney disease, *ESKD*, end-stage kidney disease, *HF* heart failure, *RAASi* renin-angiotensin-aldosterone system inhibitor.
